# Altos Labs and the quest for immortality: but can we live longer right now?

**DOI:** 10.18632/oncoscience.552

**Published:** 2022-04-22

**Authors:** Mikhail V. Blagosklonny

**Affiliations:** ^1^Roswell Park Cancer Institute, Buffalo, NY 14263, USA

**Keywords:** aging, longevity, lifespan, geroscience, rapalogs, gerostatics

## Abstract

Some visionaries prefer to dream of immortality rather than to actually live longer. Here I discuss how combining rapamycin with other modalities may let us live long enough to benefit from future discoveries in cellular reprogramming and what needs to be done at Altos Labs to make this happen.

People do not actually need to live longer to be happy; they just need to believe that they will live longer to be happy. After death, we will not know that we did not live longer anyway. Therefore, instead of using the available life-extending drug rapamycin right away, we may choose to wait for miracle discoveries.

If we believe in immortality, we do not need to be immortal. The essence of any religion is immortality in various forms, and this is what gives the meaning of life to true believers. While only a few can be true religious believers, we can all believe in scientific miracles. For this, we should be familiar with news headlines but not too familiar with the technical details. The devil is in the details. For example, headlines announce:

Jeff Bezos Is Paying For a Way to Make Humans Immortal
How To Reverse Ageing and Stay Young Forever
Meet Altos Labs, Silicon Valley’s latest wild bet on living forever
Juan Carlos Izpisua: ‘Within two decades, we will be able to prevent aging’.

The work by Juan Carlos Izpisua Belmonte, an Institute Director at Altos Labs, Inc., is brilliant. It uses genetically modified mice that express the Yamanaka factors [1, 2]. But we cannot genetically modify humans. For that, we would need a time machine to genetically modify our parents or grandparents and then to breed them in the right order to mimic mice that express Yamanaka factors. And then we would need a time machine second time to go to the future to evaluate the effect on others in clinical trials, before modifying our own grandparents.

Regarding ground-breaking studies by Juan Carlos Izpisua Belmonte and co-workers in mice [1, 2]. Although lifespan was not measured in these studies, long-lived genetically engineered mice will probably be created in the near future. Scientific discoveries cannot be predicted, otherwise the discovery has already been made at the moment of prediction. Medical applications can be expected to mainly treat trauma-related conditions [1, 3–5].

I like the science of cellular reprogramming, because it implicitly rejects the dogma that aging is caused by the accumulation of molecular damage, such as DNA damage [6]. This is in line with hyperfunction theory of quasi-programmed aging [7, 8].

Can a combination of small molecules do the same thing as Yamanaka factors? This is possible [9]. But this will be a stroke of extraordinary luck because there is no reason why such molecules should exist, and (if they exist) they have no off-target effects and are not toxic. And we still do not know whether partial reprogramming can extend normal lifespan.

Fortunately, mice have already been genetically modified to live longer by knocking out certain genes upstream and downstream of the IGF-1/mTOR signaling pathway. In these long-lived mice, the activity of the mTOR pathway is decreased [10–15]. It is extraordinarily lucky that a small molecule that inhibits the mTOR pathway exists. And, what is even more extraordinary is that this molecule has been approved for human use since 1999 for organ transplantation. As reviewed in 2007: “one target, mTOR itself, stands out, simply because its inhibitor (rapamycin) is a non-toxic, well-tolerated drug that is suitable for everyday oral administration” [16].

As proposed in 2006: “rapamycin, is already approved for clinical use, available and can be used immediately … rapamycin will be most useful as [an] anti-aging drug to slow down senescence and to prevent diseases” [7].

Starting in 2009, rapamycin was shown to extend lifespan in all species tested, in dozens of strains of mice, at various doses and schedules and was effective when given transiently and started at old age [17–24]. By slowing aging, rapamycin delays age-related diseases and especially cancer [25].

Rapamycin is now taken by an uncountable number of relatively healthy individuals, off-label, to treat aging and, by treating aging, preventing diseases. Rapamycin treatment is rapidly becoming a mainstream anti-aging intervention [26].

But what does all this have to do with Altos Labs?

First, potential life-extension with rapamycin may allow us to win time while awaiting future discoveries that will reverse aging. Figuratively, we need to slow time before reversing it; as the title queries, “Does rapamycin slow down time?” [27].

Second, rapamycin alone is unlikely to extend lifespan sufficiently to benefit from Altos Lab’s future discoveries in our lifetime. If Altos Labs would allocate a small percentage of its funding to develop rapamycin-based drug combinations, then additional decades of life extension may be available 3–5 years from now. (The same is applicable to Calico Labs).

There are three overlapping groups of drugs for rapamycin-based combinations:

Group 1: A drug (e.g., metformin, aspirin, angiotensin II receptor blockers, and PDE5 inhibitors) that is useful in several age-related diseases and conditions [28].
Group 2: Drugs that can extend lifespan in mice: Acarbose, 17-α-estradiol, and nordihydroguaiaretic acid [29–31].
Group 3: Gerostatics. In cell culture, gerostatics slow down time (figuratively), decelerating both cellular mass growth, cell cycle progression and conversion to senescence, a process known as geroconversion [32, 33]. (Note: gerostatics should not be confused with senolytics [32]). Rapamycin is a prototypic gerostatic [32, 33]. Gerostatics exert static effects on cell proliferation. In nonproliferating cells, gerostatics decelerate geroconversion. In cell culture, mTOR inhibitors (e.g., rapamycin) may increase cellular reprogramming, potentially by preventing cell senescence [34, 35]. The following gerostatics have been identified in cell culture: nutlin-3a, pan-mTOR inhibitors (such as Torins) and inhibitors of Mek, PI3K and S6K [33].

The number of potential combinations with rapamycin is enormous. All of them cannot be tested in mice. Yet, all of them do not need to be tested. I estimate the number of most important combinations as 200–300, for example, a combination of high doses of rapamycin and low doses of a pan-mTOR inhibitor [36] and/or low doses of mdm-2 and Mek inhibitors and various doses of common drugs such as metformin, acarbose, angiotensin II receptor blockers, aspirin and PDE-5 inhibitors. The full list of potential anti-aging combinations with rapamycin is beyond the scope of this editorial.
